# Generic quality of life assessment in dementia patients: a prospective cohort study

**DOI:** 10.1186/1471-2377-10-48

**Published:** 2010-06-20

**Authors:** Claudia Schiffczyk, Barbara Romero, Christina Jonas, Constanze Lahmeyer, Friedemann Müller, Matthias W Riepe

**Affiliations:** 1 Department of Psychiatry and Psychotherapy II, Mental Health & Old Age Psychiatry, Ulm University, Ulm, Germany; 2 Alzheimer Therapiezentrum Bad Aibling, Bad Aibling, Germany

## Abstract

**Background:**

Quality of life (QoL) is increasingly used to characterize the impact of disease and the efficacy of interventions.

**Methods:**

Prospective cohort study in patients' and proxies' homes with137 patients with dementia (age 52 to 88; Mini-Mental Status Examination (MMSE) 3 to 28) and their proxies (age 43 to 90). MMSE, Behave-AD, Geriatric Depression Scale (GDS), and Bayer-ADL scale (B-ADL), and the Euroqol (EQ-5D; patient self-rating, proxy self-rating, and proxy-rating of patient).

**Results:**

B-ADL impairment and Behave-AD total score increased with dementia severity (Kruskal-Wallis p < 0.001 and p = 0.023, respectively). Patients' self-rated QoL and proxies' self-rated QoL were unrelated to dementia severity (p = 0.148 and p = 0.414, respectively). The difference between patients' self- and proxies'-rating of the patient's QoL correlated with the patient's MMSE (Spearman's rho = -0.434; p < 0.001), even if analysis was constrained to patients with mild AD (rho = -0.328; p = 0.019). The proxies' rating of the patients QoL was not only correlated with cognitive and behavioral symptoms of the patient but also with mood (GDS-score; rho = 0.317; p < 0.001) and cognitive abilities (verbal fluency; rho = 0.209; p < 0.018) of the proxy.

**Conclusion:**

Proxies' assessment of the patients' QoL is related to the proxies' health, and the difference of patient's and proxie's QoL-rating is correlated with dementia severity even in mild dementia stages. QOL measures use ratings of the individual to assess the impact of symptoms and disorders on everyday life. In dementia patients, however, this impact is not captured since patients' and proxies' self-assessment of their own QoL do not reflect severity of disease whatsoever. Patients' and proxies' influencing variables render the score obtained with generic quality of life assessment meaningless in capturing the impact of dementia. Decisions on initiation or discontinuation of treatment or allocation of other resources for patients with dementia therefore need not depend on generic assessment of quality of life.

## Background

Dementia is a frequent disorder in the elderly and its prevalence increases with age [1]. Due to the demographic change, the incidence and prevalence of dementia and the number patients and their proxies will increase in the coming years. This challenges the health care system to provide effective treatment under the constraints of limited economic resources.

The Constitution of the World Health Organization (WHO) defines health as "A state of complete physical, mental, and social well-being not merely the absence of disease. . .". From this definition it is concluded that measurement of health and the effects of health care must include not only an indication of changes in the frequency and severity of symptoms but also an estimation of the well-being by measuring the improvement in the quality of life (QoL) related to health care. QoL is expected to convey greater meaning and more direct relevance across a wide spectrum of diseases and illnesses than clinical scales or instruments. Assessment of the QoL also aims at achieving comparability of the burden of diseases across the broad array diseases in different specialties. The WHO summarizes that QoL-instruments have many uses, including use in medical practice, research, audit, and in policy making.

QOL measures use the subjective ratings of the individual in a variety of areas to assess the impact of symptoms and disorders on life [2]. By this definition Qol is a subjective construct, being evaluated and self-reported by the affected person. In a similar fashion, the influential model by Lawton characterized five domains pertaining to QoL for subjects with dementia to comprise the same areas as for people in general (cognitive functioning, ability to perform activities of daily living, being able to engage in meaningful time use, social behavior, and a favorable balance between positive emotion and absence of negative emotion) [3]. Similar to the WHO concept this model is predominantly self-evaluated and self-reported, although some objective elements, such as perceived contentment and functional abilities are supplemented. Several instruments have been developed, with the Euroqol being one of the most widely applied [4-7]

Previous studies on the validity of the patient self-rating of the quality of life are contradictory. It has been reported that patients with Alzheimer's dementia are likely to give overly optimistic ratings of their own mental capacities, their functions, activities and social relationships [8]. Several studies, however, report that the capability of self-rating of Qol is not impaired by the severity of cognitive impairment [9-12].

The use of proxies (e.g relatives or nurses) to measure QoL has inherent obstacles, such as personality characteristics of the proxy, the nature of the relationship, the time spent with one another, and the level of impairment. Frequently, proxy appraisal of the patient's QoL are disparate to the patients own evaluation [13] but it was interpreted that patient- and proxy report may both represent valid, although differing, perspectives on quality of life [14]. Discrepancies between ratings of dementia patients' and their proxies' are reported to be associated with increased levels of caregiver burden, rather than lower levels of patients' functioning alone [15,16]. The proxie's experiences of depression and burden might also negatively affect proxies' assessments of QoL [13].

For this study a large number of patients and proxies were interviewed in their homes with an extensive battery comprising the Mini-Mental-Status Examination (MMSE), the cognitive scale Alzheimer's disease assessment scale (ADAScog), the Behavioral pathology in Alzheimer's Disease Rating Scale (Behave-AD), the Geriatric Depression Scale (GDS), the Bayer-Activities of daily living scale (B-ADL). It was the goal to assess the self- and proxy-ratings of QoL and their relation to the severity of AD.

## Methods

The study was performed according to institutional guidelines and the principles laid out in the Declaration of Helsinki. Written informed consent was obtained by patients and proxies.

### Patients and Caregivers

The patients and their proxies were recruited from a cohort of patients applying for a short-term in-patient treatment at the Alzheimer Therapy Center Bad Aibling. Initial contact and screening of the eligibility to take part in the study were made via telephone by a study nurse. Criteria for inclusion in the study were a diagnosis of dementia of mixed or Alzheimers type performed by a general practitioner or neurologist/psychiatrist. Only patients living in one household with their primary proxy were included in the study. Current analysis of this ongoing study included all patients with baseline assessment between September 2008 and December 2009 with a MMSE score of 3 and above being able to complete the Geriatric Depression Scale. Six patients had to be excluded for not being able to complete the GDS scale.

The remaining sample comprises 137 patients with either AD or mixed dementia (mean age 73.0 ± 6.7 years, range 52 - 88 years; 69.3% male) and their proxies (mean age 69.6 ± 7.6 years, range 43 - 90 years; 70.1% female; 98.6% spouses). MMSE scores ranged from 3 - 28 (mean 16.9 ± 6.4). The basic demographic variables are described in Table 1.

**Table 1:** Demographic data of study participants.

	Patients	Proxies
**n**	137	137
		
**Age (median)**	74	70
**Age (range)**	52 - 88	43 - 90
		
**Male (%)**	69.3	29.9
**Female (%)**	30.7	70.1
		
**MMSE (median)**	17.0	
**MMSE (range)**	3 - 28	

MMSE: Mini-Mental-Status Examination (score 0 - 30).

All interviews took place in the domestic surroundings of the families after explaining the aim of the study and obtaining informed consent by both the patient and the proxy. Assessments were carried out by specially trained research assistants. Patients and proxies were interviewed separately to minimize bias and mutual influence on the responses.

### Assessments

#### Mini-Mental Status Examination (MMSE [17])

The MMSE is the most commonly used instrument to stage the severity of dementia by assessing cognitive functions. It comprises tests on orientation, registration, short-term memory, language use, comprehension, and basic motor skills. The score ranges from 0 - 30. Commonly, the scores on the Mini-Mental Status Examination are used to describe the severity of dementia. Patients are considered to be in mild stages of disease when scoring 20 points or above, to be in moderate stages of disease when scoring between 10 and 19, and severe when scoring 9 or less.

#### Semantic fluency [18]

Assessment of the semantic fluency is a measure of executive functioning. It can be used to screen for cognitive deficits [19] and was used in this study to assess proxies.

#### Behavioral pathology in Alzheimer's Disease Rating Scale (Behave-AD [20])

The Behave-AD is a clinical rating instrument to characterize the phenomenology of behavioral symptoms. It comprises 25 items, all of which are answered by the proxy.

#### Geriatric Depression Scale (GDS [21])

The Geriatric Depression scale uses a 15-item questionnaire to assess symptoms of depression and has been validated in cognitively intact and demented elderly [22,23].

#### Activities of Daily living (Bayer-ADL [24])

This scale is used to assess the deficits in the performance of the patients' everyday activities. It comprises 25 items, all of which are answered by the proxy. Ratings are made on a 10-point Likert-type scale.

#### Euro-QoL (EQ-5D [25])

The EQ-5D questionnaire is a generic instrument to measure health related QoL in five domains: mobility, self-care, usual activities, pain/discomfort and anxiety/depression. It can be applied to patients as well as used in proxies to rate their own and the patients' QoL [26,27]. There are two core components of this instrument: a description of the respondents own health in the above mentioned five domains (rated on a three point Likert scale each) and a rating of the overall own health on a visual analog scale (VAS, score 0 - 100). For the present study the VAS was not analyzed. In order to capture the cognitive deterioration, a cognitive dimension was added (EQ-5D + C). This allows to calculate the scores for the EQ-5D and te EQ-5D + c. In the present study both the patient and the proxies completed the EQ-5D + C (patient-self-rating and proxy-self-rating, respectively). Additionally, the proxy was asked to rate the quality of life of the patient: proxy-patient-rating. Since the interviews were carried out separately, patients and the proxies had no opportunity to distort rating by exchanging their answers.

### Data Analysis

All statistical analyses for the investigation of group differences were carried out using the statistics program SPSS (SPSS 15.0 for Windows, Chicago, Ill., 2001). The normality of the distribution was tested with the Kolmogorov-Smirnov Test. As the data were not normally distributed we used the Wilcoxon-test and the Spearman's correlation coefficient.

## Results

### Disease severity - cognition, mood, and activities of daily living

Commonly, the severity of dementia is grouped according to the scores in the MMSE. Accordingly, we analyzed the relation between quality of life assessments and the severity of disease (mild: MMSE score ≥20; moderate: 10 ≤ MMSE score ≤19; severe: 3 ≤ MMSE score ≤9). The study included 51 patients with mild, 66 patients with moderate and 20 patients with severe dementia.

Proxies of patient that are more severely affected report more behavioral symptoms (BEHAVE-AD; p = 0.021) and more impairment in activities of daily living (Bayer-ADL; p < 0.001). Patients themselves, however, do not report an increase of depressive symptoms (GDS; p = 0.985).

### Disease severity and quality of life

Severity of dementia is not mirrored in the self-rating of the quality of life of the patient and the self-rating of the quality of life of the proxy. The results of the one-factorial Kruskal-Wallis test for the self-rating of QoL of the patient, the proxy-rating of the QoL of the patient, and the proxy-patient-rating of the QoL for the EQ-5d and the EQ-5d with additional cognitive domain are shown in Table 2.

**Table 2:** Dementia severity and patient self-rating of the quality of life, proxy self-rating of the quality of life, and proxy-rating of the patients' quality of life.

	EQ-5d	EQ-5d + c
**Proxy self-rating**	0.414	0.392
**Proxy rating of patient**	0.002	0.001
**Patient self-rating**	0.148	0.088

p-values from Kruskal-Wallis test for comparison across mild, moderate, and severe dementia. EQ-5D: Euroqol. EQ-5D + C: EQ-5D plus cognitive domain.

In the self-assessment of both the patient and the proxy no differences were found in the EQ-5d or the EQ-5d with additional cognitive domain. However, the proxy assessment of the patients' quality of life decreased with increasing severity of dementia of the patient.

### Patient-rating and proxy-rating

**Figure 1:**
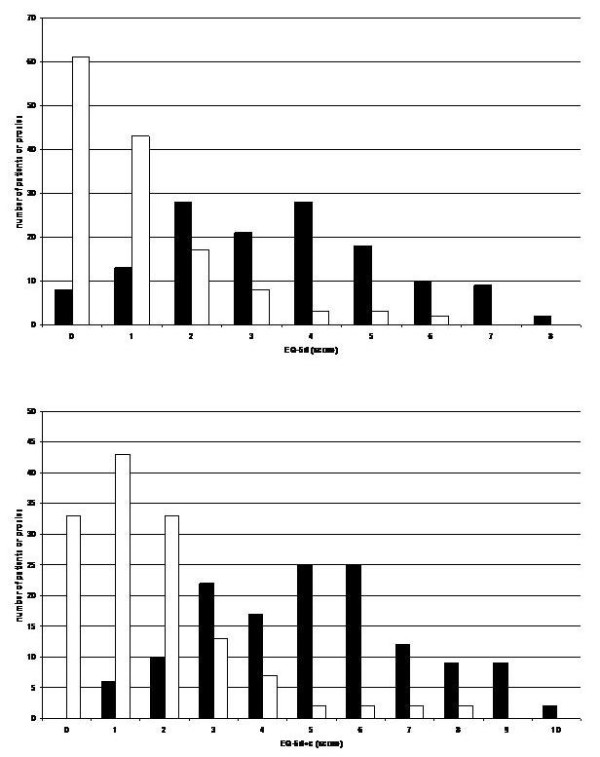
Scores in the A) Euroqol (EQ-5d; score range EQ-5d: 0 - 15) and the B) Euroqol with added cognitive domain (EQ-5d + c; score range EQ-5d + c: 0 - 18) for the patient self-rating (white bars) and the proxy-rating of the patient (black bars).

In both the EQ-5d and the EQ-5d with supplemented cognitive domain a discrepancy between the judgment of the proxy and the judgment of the patient is observed (Figure 1).

**Figure 2:**
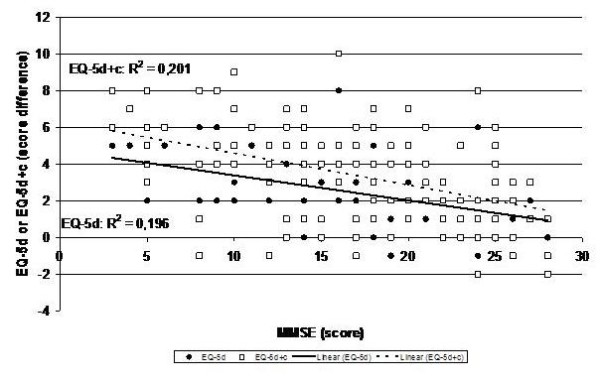
Difference of the proxy-rating of the patients' quality of life and the patient self-rating for the EQ-5d (black dots; score range EQ-5d: 0 - 15) and the EQ-5d + c (white squares; score range EQ-5d + c: 0 - 18) against the MMSE score (score range MMSE: 0 - 30).

The difference between the patients estimation of QoL and the proxie's estimation of the patient's QoL correlates with the MMSE of the patient (Spearman's rho EQ-5d = -0.434; rho EQ-5d + c = -0.444, respectively; p < 0.001 both). Even if the analysis is confined to patients with an MMSE score of 20 and above the difference between the patients estimation of QoL and the proxie's estimation of the patient's QoL correlates with the MMSE of the patient (Spearman's rho EQ-5d = -0.328 (p = 0.019); rho EQ-5d + c = -0.347 (p = 0.012) (Figure 2).

Estimation of the patient's QoL by the proxy correlates not only with patient-related parameters but also with the GDS-score of the proxy (EQ-5d: Spearman's rho = 0.317, p < 0.001; EQ-5d + c: rho = 0.336, p < 0.001) and cognitive functions such as the semantic fluency (EQ-5d: Spearman's rho = 0.209, p < 0.018; EQ-5d + c: rho = 0.216, p < 0.014). Thus, proxies that are more depressed and/or have better performance in the semantic fluency task themselves rate the quality of life of the patient worse.

## Discussion

With progressing severity of dementia cognitive abilities of the patient, behavioral symptoms and activities of daily living are impaired. This implies that the patient's autonomy is reduced. Neither is the patient able to uphold leisure activities on his or her own nor is the patient able to sustain functions to preserve living such as household chores or food preparation without help. In the EQ-5d two questions specifically ask for the ability to care for oneself and everyday activities. Patients' interpretation and self-rating of these questions, however, may deviate from objective capturing of measures of these variables and may be more subjectively interpreted to also depend on other variables such as the burden of proxies resulting in differences in the availability of care for the patient. Therefore the term 'quality of life' may not mean the same thing when patients and healthy persons talk about it. Thus, the interpretation of 'quality of life' likely remains ambiguous even when patients and proxies are asked the same questions.

In contrast to previous investigations, the present study was performed with visitation of patients and their proxies in their household to catch their assessment in their familiar environment. There are some possible limitations to this approach. Although the participants were recruited country-wide this cohort may represent a subsample of the population as the proxies and patients were recruited from a database of patients that had applied for a short-term rehabilitation program in a specialized dementia service (Alzheimer Therapy Center Bad Aibling, Germany). In contrast to other studies, however, assessment of the patients and proxies in their familiar surroundings excludes distortion by being asked in a less well known environment such as a medical office.

A lack of deterioration of quality of life assessment with current instruments in more severe stages of disease was interpreted in a way that the actual life's quality of the patients does not deteriorate with severity of disease [12]. This rests on reports that the capability of self-assessment of Qol is not impaired by the severity of cognitive impairment [9,10,28]. Other studies, however, concluded that the validity of self-reported QoL is uncertain in AD [29-32] because patients in early stages of AD are likely to give overly optimistic ratings of their capabilities and activities [8]. The present study demonstrates that in spite of deterioration of cognitive abilities, behavior, and the impairment of daily functions an established instrument to assess the quality of life does not pick up this loss of function. The self-reported quality of life of patients does not decrease in more severe stages of dementia. One reason may be, that patients with dementia often have decreased awareness of their cognitive impairments and changes in behavior [8,33,34] which likely results from affliction of brain regions partaking in awareness of functional capacities with increasing severity of dementia [35]. An alternative explanation is that patients with dementia receiving support by caring proxies do not perceive limitations in their life. However, this chain of thought implicitly presumes the patient to be able to value his or her situation and abilities. Applying Ockham's principle of parsimony we conclude on neurobiological grounds and the results of the present study with patient's self-assessed quality of life scales not reflecting obvious everyday impairments whatsoever and proxy-assessment of the patient being dependent on proxies' mood and cognitive abilities that QoL instruments are useless in dementia research. The influencing variables on the side of the proxy have far-reaching implications considering the current discussion on advanced directives and end-of-life issues. When proxies are being asked on end-of-life issues on behalf of the patient they may not be able to capture the impact of disease on the patients' lifes having the concept of 'quality of life' in mind. This resembles the discussion in another neurodegenerative disease, amyotrophic lateral sclerosis, where proxies may draw other conclusions on life-sustaining treatment from assessing the medical condition of the patient than patients themselves [36].

One would expect that the quality of life of the proxy is affected in more severe stages of dementia in the patient as the proxy has to compensate for the loss of function of the patient. However, the present study shows that the increased demand on the caregiver does not result in a decreased quality of life in the self-assessment of the proxy which is similar to a previous report [10]. This may indeed reflect the successful adaptation of the caregiver or successful use of coping strategies. Potentially this may also indicate that a sizeable portion of proxies of dementia patients suffer from cognitive problems or neuropsychiatric disorders as well [37].

Other than the patients' self-assessment, the proxies' assessment of the patients' quality of life is reduced with increased dementia severity of the patient. In the present study proxies rated the Qol of the patient worse than the patient in both the conventional Euroqol as well as when the score included an additional cognitive dimension (EQ-5D + c). It has been reported, previously, that proxy-ratings of Qol in AD patients do not correlate with the patients' self-report [13,38,39]. The present study shows that the discrepancy between self- and proxy rating of QOL increases with severity of disease.

A previous study concluded that patient- and caregiver report may both represent valid, although differing, perspectives on quality of life [14]. The present study, however, demonstrates that the proxies' assessment of the patients' quality of life is not only related to the disease process in the patient but to factors of the proxy such as their mood and their own cognitive function. To further clarify these issues it would be necessary to assess additional variables such as availability and use of social services, day care centers, support groups, and also other instruments to appraise burden of disease or impact of disease. These were not included in the present study because the availability of resources and living conditions were anticipated to vary too much across the nationwide recruitment area. Future research, however, should address these issues.

## Conclusions

We conclude that rating of the QoL with the Euroqol is a useless measure in dementia research. Patients' and proxies' self-assessment of their own QoL does not reflect severity of disease. Proxies' assessment of the patients' QoL is related to the proxies' health, and the difference of patient's and proxie's QoL-ratings is correlated with dementia severity even in mild dementia stages (MMSE equal or above 20). Thus, patients' and proxies' influencing variables render the score obtained with generic quality of life assessment meaningless in capturing the impact of dementia. Therefore, decisions on initiation or discontinuation of treatment or allocation of other resources for patients with dementia therefore need not depend on generic assessment of quality of life.

## Competing interests

The present study was funded by the German Federal Ministry of Health (BMG-LTDemenz_04_61). The sponsor did not influence design of the study, analysis of the data, or drafting of the manuscript. CJ, CL, and CS report no conflict of interest. BR, FM, and MWR have received grants or funding and honoraria from companies selling or developing medicinal products for use in patients with Alzheimer's disease. None of the authors report personal or financial conflicts of interest.

## Authors' contributions

CJ, CL, and CS were involved in acquisition of the data, data analysis, and drafting and revising the manuscript. BR, FM and MWR were involved in designing the study, interpretation of the data, and drafting and revising the manuscript. All authors approved the final version of the manuscript.

## Pre-publication history

The pre-publication history for this paper can be accessed here:

http://www.biomedcentral.com/1471-2377/10/48/prepub
